# Advances in Sorptive Removal of Hexavalent Chromium (Cr(VI)) in Aqueous Solutions Using Polymeric Materials

**DOI:** 10.3390/polym15020388

**Published:** 2023-01-11

**Authors:** Xiaoqing Yuan, Jingxia Li, Lin Luo, Zhenyu Zhong, Xiande Xie

**Affiliations:** 1 College of Resources and Environment, Hunan Agricultural University, Changsha 410128, China; 2 Hunan Research Academy of Environmental Sciences, Changsha 410014, China

**Keywords:** polymeric materials, hexavalent chromium, sorptive removal, trivalent chromium, chromium redox

## Abstract

Sorptive removal of hexavalent chromium (Cr(VI)) bears the advantages of simple operation and easy construction. Customized polymeric materials are the attracting adsorbents due to their selectivity, chemical and mechanical stabilities. The mostly investigated polymeric materials for removing Cr(VI) were reviewed in this work. Assembling of robust functional groups, reduction of self-aggregation, and enhancement of stability and mechanical strength, were the general strategies to improve the performance of polymeric adsorbents. The maximum adsorption capacities of these polymers toward Cr(VI) fitted by Langmuir isotherm model ranged from 3.2 to 1185 mg/g. Mechanisms of complexation, chelation, reduction, electrostatic attraction, anion exchange, and hydrogen bonding were involved in the Cr(VI) removal. Influence factors on Cr(VI) removal were itemized. Polymeric adsorbents performed much better in the strong acidic pH range (e.g., pH 2.0) and at higher initial Cr(VI) concentrations. The adsorption of Cr(VI) was an endothermic reaction, and higher reaction temperature favored more robust adsorption. Anions inhibited the removal of Cr(VI) through competitive adsorption, while that was barely affected by cations. Factors that affected the regeneration of these adsorbents were summarized. To realize the goal of industrial application and environmental protection, removal of the Cr(VI) accompanied by its detoxication through reduction is highly encouraged. Moreover, development of adsorbents with strong regeneration ability and low cost, which are robust for removing Cr(VI) at trace levels and a wider pH range, should also be an eternally immutable subject in the future. Work done will be helpful for developing more robust polymeric adsorbents and for promoting the treatment of Cr(VI)-containing wastewater.

## 1. Introduction

Chromium and its compounds are widely used in a variety of industries, such as electroplating, tanning leather, printing, and dyeing [1], making it the major heavy metal pollutant in industrial wastewater. Chromium commonly exists in trivalent (Cr(III) and hexavalent (Cr(VI)) states in aqueous solutions [2]. Cr(VI) is a top-priority pollutant and it is much more toxic and mobile than Cr(III) [3]. Removal of Cr(VI) from wastewater is in urgent need for protecting the safety of aquatic environment. Because of the advantages of simple operation and easy construction, removal of Cr(VI) using the natural and synthetic adsorbents has become one of the most preferred choices [4]. An ideal adsorbent is expected to have high selectivity and affinity toward Cr(VI), and at the meantime, it should be nontoxic, cheap, and readily available [5]. However, most easily available natural adsorbents (e.g., clay, metal hydroxides, chitosan, and cellulose) were hard to realize this target due to their insufficient adsorption ability, weak mechanical and thermal stability, and difficulty in regeneration [6]. Thus, the development of a more efficient Cr(VI) adsorbent is still an exigent mission.

**Figure 1:**
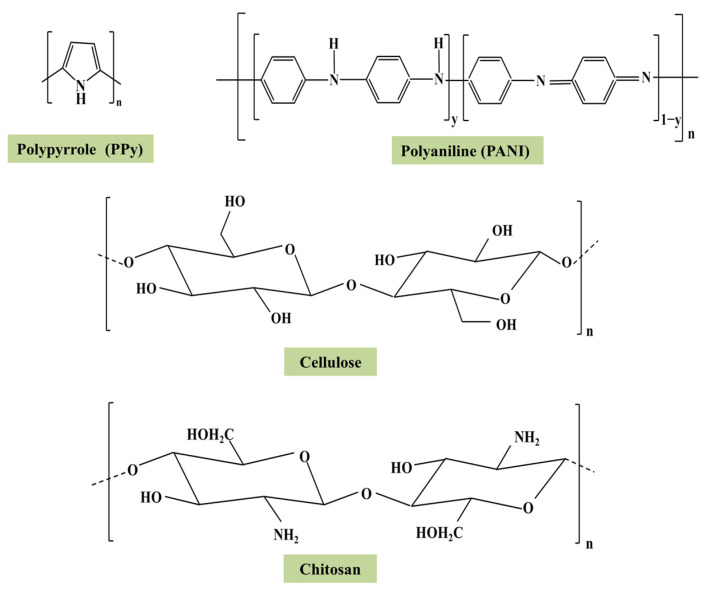
The chemical structures of several selected polymers. Reproduced from Refs. [22,23,24].

Bearing the merits of low-cost, large surface area, high selectivity, and high adsorption capacity, polymeric materials have attracted increasing attention in removing Cr(VI) [5], which can be reflected by the sharply increasing papers published in this field (more than 300 papers in recent decades). Polypyrrole (PPy), polyaniline (PANI), polysaccharides, chitosan, and cellulose have been applied for removing Cr(VI) in wastewater [7,8,9,10,11] (Figure 1). Mechanisms of ion exchange, electrostatic attraction, ligand exchange, reduction, chelation, and hydrogen bonding were involved in Cr(VI) removal. The exact driving forces of Cr(VI) removal depended on the physicochemical properties of polymers. For example, polymers rich in amine, imine, and catechol functional groups are capable of reducing Cr(VI) to Cr(III) to increase its removal [12]. Self-agglomeration, which might result in the reduction of active sites and difficulty of reuse, is an obstacle for industrial application of polymers [13]. Thus, considerable researches have focused on the modification of the surface structures of polymers to minimize the self-agglomeration and assemble more robust functional groups to enhance the adsorption of Cr(VI) and to simplify the reuse of these materials [14,15]. Strategies, including coating on the other substrate materials [13], reconstructing the structure of polymers through crosslinking and hybridization [14,16], grafting functional groups and branched chains [17], and acting as a scaffold to combine with other absorbents [18], are applied. For example, PPy coated molybdenum disulfide organic–inorganic composites exhibited much higher sorption capacity for Cr(VI) than neat PPy (257.7 vs. 151.3 mg/g) [19]. Loading of PANI onto the neodymium-doped Zn-Al layered double hydroxide dramatically enhanced the sorptive removal of Cr(VI) compared to the raw layered double hydroxide [20]. Meanwhile, the maximum adsorption capacity of core/shell-like alginate@ polyethylenimine composites for Cr(VI) was calculated to be 497.1 mg/g based on Langmuir isotherm at the concentration of 0–600 mg/L under pH 2.0 [21]. Despite the rapid progresses in this field, there is lack of a comprehensive overview to conclude the work done, not done, and need to be done. Therefore, a systematic review covered the newly published papers is in urgent need to indicate the direction of designing more effective adsorbents and to promote the industrial application of these newly designed materials.

Herein, the recent progress in designing and applying polymeric materials for removing Cr(VI) is systematically reviewed. The physiochemical properties and their performance in removing Cr(VI) are compared with each other. The mechanisms of these newly designed polymeric materials are delineated, and the differences between these sorbents are analyzed. The influencing factors that affected the adsorption are also highlighted. Finally, the challenges and perspectives for capturing Cr(VI) using polymeric materials are proposed. Work done will help in the development of cost-effective sorbents for removing Cr(VI)-containing wastewater.

## 2. Polymers Used for Cr(VI) Removal

### 2.1. Polypyrrole (PPy)-Based Adsorbents

**Figure 2:**
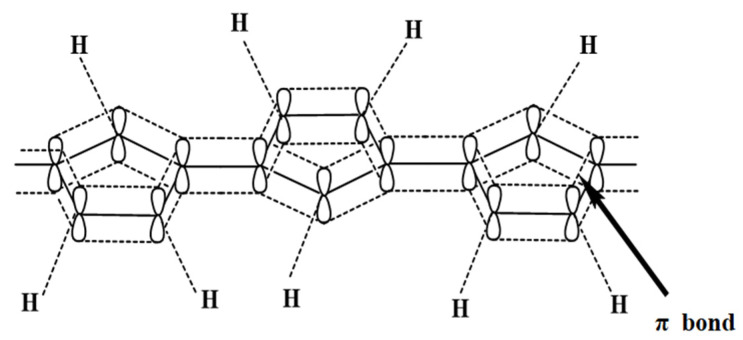
The π bond of ppy. Reproduced from Ref. [37].

Chigondo et al. had previously reviewed the removal of Cr(VI) using PPy-based adsorbents [7]. They summarized the removal efficiencies of varied PPy adsorbents, and the mechanisms of Cr(VI) removal and methods for the regeneration of these PPy-based adsorbents were also reported [7]. Bearing the merits of reputable environmental stability, extraordinary conductance, desirable redox properties, ease of processing, and rich in amine groups, PPy has shown great prospects in sorptive removal of Cr(VI) (Table 1). The positively charged nitrogen atoms in the polymer chain of PPy are capable of binding Cr(VI) to facilitate its adsorption, and at the meantime resulting in the reduction of Cr(VI) to Cr(III) [25]. Both effects facilitate Cr(VI) removal. However, self-agglomeration often occurs in pure PPy due to the strong π-π interactions between the main chains (shown in Figure 2), making it difficult to disperse in water phase, which in turn results in the decrease of its surface area and adsorption capacities [26]. Moreover, separating the PPy powder from the reaction mixture is also challenging [27]. To counteract these adverse effects, strategies of innovating synthesis method to modify the surface properties of PPy and developing composites are applied. Except for bulk PPy powder, free standing PPy films at the air/water interface were also formed during the rapid mixing of the pyrrole monomer and FeCl_3_ [28], which increased the positive surface charge density on PPy surface and thus reduced the agglomeration when compared to the method of dropwise addition of PPy monomer to FeCl_3_ solutions. As a consequence, the Cr(VI) removal efficiency increased from 40.2% to 69.5% [28]. Meanwhile, PPy-based magnetic materials were also prepared to enhance the recovery of PPy [29]. The surface PPy functionalized sepiolite fiber was prepared by assembling PPy on the sepiolite fiber surface, which had features of magnetism and floatation [26]. Thus, this newly prepared adsorbent can be easily recovered from the waterbody through magnetic field [26], and the maximum adsorption capacity toward Cr(VI) at concentrations of 10–100 mg/L achieved 108.9 mg/g obtained by Langmuir isotherm model at pH 2.0 and 25 °C [26]. The synthesis of PPy-based composites using biochar [30], molybdenum disulphide [31], clay [32], cellulose [33], and graphene oxide [34] were also been documented in present literatures. The mechanisms of enhanced Cr(VI) removal involved in the above PPy-based composites were also the reduction of agglomeration and the increase of active sites. As shown in Table 1, these PPy-based adsorbents have Brunauer–Emmett–Teller (BET) surface areas (SBET) of 15.9–149.8 m^2^/g, and exhibit the maximum adsorption capacity toward Cr(VI) in the range of 103.6 to 961.5 mg/g (Table 1). The isoelectric point of the PPy-based absorbents was 2.3–10.2 and the optimum pH mostly occurred at ~2.0 (Table 1). The redox potential of Cr(VI) decreases with the increasing pH, and Cr(VI) mainly exists in the anionic form in solution [35]. The isoelectric point of PPy is at pH ~ 4.3, and it carries positive charges on its surface in pH < 4.3 solution [36]. The enhanced reduction of Cr(VI) to Cr(III) and the increased electrostatic attractions between chromium and PPy-based adsorbents jointly contributed to Cr(VI) removal at pH ~ 2.0 [35]. Except that the maximum adsorption generally occurred in the strong acid medium, the oxidation of these PPy-based adsorbents might result in the rupture of the polymer chain to reduce the adsorption [7] (Table 1). Therefore, more efforts are necessary to minimize the structural deterioration of PPy-based adsorbents and acid consumption during the removal of Cr(VI).

### 2.2. Polyaniline (PANI)-Based Adsorbents

The performances and mechanisms of Cr(VI) removal using various PANI modified adsorbents had been summed up previously by Jiang et al. in 2018 [8]. Polyaniline (PANI) is an attractive material which has been widely used for Cr(VI) removal due to its controllable electrical conductivity, excellent redox properties, low price, and simple doping (Table 1) [38]. PANI has both amine and imine groups in its structure, thus it exhibits the reductive potential of Cr(VI) as that of PPy [39]. The ratios of amine and imine functional groups on PANI make it present three oxidation states, i.e., leucoemeraldine (LB), emeraldine (EB), and pernigraniline (PB) [40]. The oxidation states shift from LB to EB and PB during the reduction of Cr(VI) by PANI [41]. The removal of Cr(VI) by PANI is highly affected by solution pH due to the protonation and deprotonation of nitrogen atoms [42]. It is well documented that the redox potential of Cr(VI) increases with the decrease of pH [35], which makes it easier to be reduced to Cr(III) at lower pH. Meanwhile, the general bulky PANI prepared by oxidative polymerization had an isoelectric point of 7.3 [43], and it was positively charged in pH < 7.3 solutions. Electrostatic attraction between the positively charged PANI and Cr(VI) anions could enhance the removal of Cr(VI) in acidic environment [44]. Therefore, the optimum pH used for Cr(VI) removal by PANI was typically in the acidic range [45]. Furthermore, PANI has high resistance against acidic conditions and only dissolves in concentrated sulfuric acid [46]. Despite of these merits of PANI, the specific surface area of the normal bulky is typically low (22.4–31.7 m^2^/g) to provide sufficient adsorption and reaction sites [43,47]. Besides, the mechanical strength of these bulky PANI powders is low [48]. The ultrafine PANI particles have relatively high specific surface area, while they are hard to be recycled and used directly in flow-through systems [48].

To overcome the adverse effects of PANI alone, PANI composites were synthesized [49]. Preparation of PANI into a well-organized hollow spherical structure using poly(styrene-co-acrylic acid) spheres as a templet dramatically increased its specific surface area and recyclability [50]. The maximum adsorption capacity of Cr(VI) by the hollow spherical PANI reached 601.3 mg/g at pH of 1.0 solution with an initial Cr(VI) concentration of 0–100 mg/L [50]. The maximum removal capacity of Cr(VI) by porous PANI synthesized with the bacteria cell as templates was calculated to be 835.06 mg/g at concentrations of 1–1500 mg/L at pH 1.0 [51]. In addition, more than 90% of Cr(VI) could be reduced to Cr(Ⅲ) by bacteria cell templated porous polyaniline in both acidic and neutral solutions [51]. Coating of PANI on sawdust [52], fibers [53], graphene oxide [54], and inorganic complexes [55], were also documented to enhance the removal of Cr(VI) by increasing the specific surface area [48], the treatment pH [56], hydrophilicity of adsorbents, and enhancing the reduction of Cr(VI) to Cr(III) [13]. It is worth noting that some PANI-based adsorbents showed excellent adsorption performance of Cr(VI) in neutral or nearly neutral solutions and real water. Polyaniline confined in the pores of polystyrene beads presented an adsorption capacity of 233.7 mg/g at near neutral pH (6.0) [48]. The Cr(VI) (initially at 200 mg/L) removal efficiency by polyaniline coated bacterial cellulose (PANI/BC) mat reached higher than 99% at pH 7.0 within 24 h [56]. UiO-66 derived N-doped carbon nanoparticles coated by polyaniline (PANI@NC-600) exhibited similar treatment performance of removal in deionized water as that of in Dushu Lake and Jinji Lake water, indicating the robust ability to counteract the adverse effect brought by water constituents [39]. However, the coated PANI might come off from the composite structure during use of these adsorbents which hindered the reuse of these composites. Moreover, the synthesis of these PANI composites is required to operate in strictly controlled conditions and time consuming (Table 1), which might increase the overall treatment cost. Therefore, more simple and convenient preparation methods are needed to prepare more robust PANI composites which are effective for Cr(VI) removal and feasible for reuse.

### 2.3. Cellulose-Based Adsorbents

Cellulose-based adsorbents have been widely used in water treatment, and their modification methods, properties, and application have been reviewed in literature [23,57,58,59]. However, the sorptive removal of Cr(VI) by cellulose-based adsorbents has not been systematically reviewed. Cellulose is the most abundant natural pappolymer, in which the D-glucose monomers are linked with β-1, 4-glycosidic bonds [24]. It can be extracted from wood and annual plants, microbes (bacteria and algae) and animals (tunicates) [60,61]. Different extraction methods result in the varied physical forms of cellulose, such as mats [56], hydrogels [62], foam [63], nanocrystals [64], and nanofibrils [65]. Rich in hydroxyl groups, cellulose shows high affinity toward heavy metals and offers suitable platform for functionalization [66,67]. The structure of cellulose can be modified through blending and grafting of functional components to increase the active sites [64,68]. The cellulose backbone grafted by the desired monomers (e.g., poly(m-aminobenzene sulfonate), and polyethylenimine), which can be further functionalized with known chelating groups (ammonium, amino, etc., Table 1). Amino-functionalized magnetic cellulose nanocomposites achieved a maximum adsorption capacity of 171.5 mg/g toward Cr(VI) of 0–250 mg/L at pH 2.0 and 25 °C, and the adsorbent was easily recovered by magnetic field [68]. Meanwhile, cellulose-based adsorbents with pendant groups of quaternary ammonium and amino for Cr(VI) removal presented a maximum Cr(VI) uptake capacity of 490.3 mg/g at concentrations of 20–700 mg/L at pH 2.0 and 30 °C [69]. Cellulose-based absorbents were reported to treat Cr(VI) at the initial concentrations of 1.0–1000 mg/L (Table 1). For example, acrylamidethiosemicarbazide cellulose aerogels were proven to effectively remove at relatively low levels (i.e., 1.0–20 mg/L) [70]. The removal rate of Cr(VI) reached 99% and the maximum effluent concentration of Cr(VI) was lower than 0.045 mg/L [70]. While, the polyaniline-impregnated nanocellulose composite could remove 84.9% of the Cr(VI) at an initial concentration of 1000 mg/L [67]. Despite that modification of cellulose has dramatically enhanced its ability to remove Cr(VI), the application of this natural polymers is still in the road to improve the adsorption efficiency, enhance the regeneration performance, and simplify the separation process (Table 1).

### 2.4. Chitosan-Based Adsorbents

**Figure 3:**
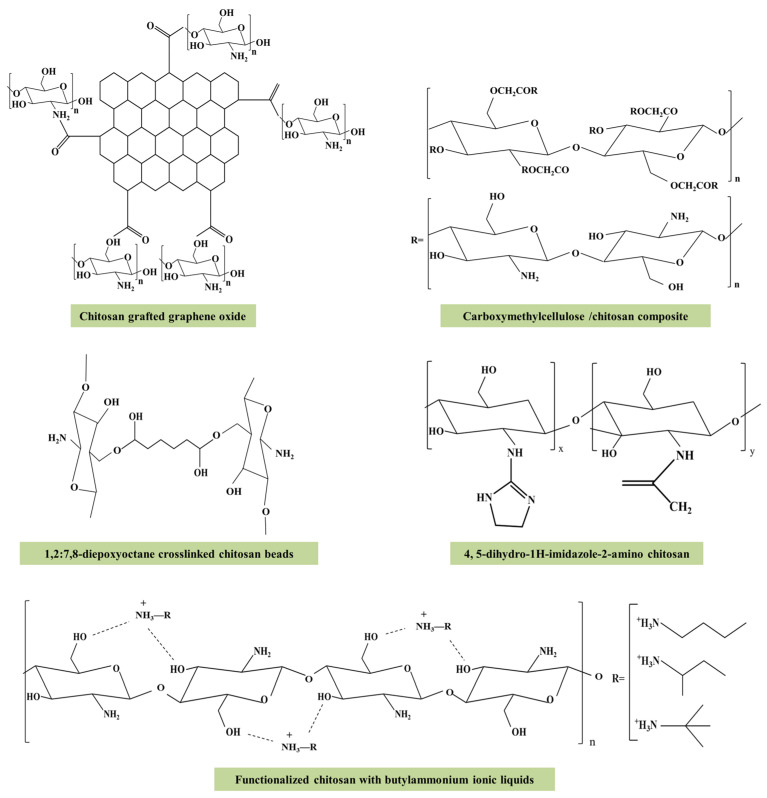
Several chemical structures of modified chitosan-based adsorbents for Cr(VI). Reproduced from Refs. [10,79,80,81,82].

Available reviews overviewed the developments of chitosan-based adsorbents in water treatments [71,72], while limited information was available for Cr(VI) removal. Chitosan, which is generated by the deacetylation of chitin, is the second most abundant natural biopolymer [73]. Chitosan has one –NH_2_ group and two –OH groups in its glucosamine units [74], which facilitate its binding to metal ions by chelation in neutral solutions and by electrostatic attraction in acidic medium, respectively [75]. It has low price and high reactivity toward a variety of contaminants [76]. However, this natural polymer is unstable in pH < 4.0 solutions, which hinders its application [73]. To strengthen the performance of chitosan, several physical and chemical modification methods have been applied [77]. Various forms of chitosan were prepared by physical modifications to increase the specific surface area and the stability of structure, including powder, nanoparticles, gel beads, membranes, sponges, type structure of “honeycomb” and various types of fiber [75]. Meanwhile, chemical modifications which involve uniting the macromolecular chains with each other via cross-linking and inserting functional groups by grafting are also the strategies to increase the adsorption sites and acid-resistance ability [71]. Figure 3 presents the structure of several functional modified chitosan-based absorbents of Cr(VI). The presence of –NH_2_ and –OH groups provided a platform for modification of chitosan. As shown in Table 1, most of the modified chitosan-based adsorbents performed well under acidic conditions. The maximum adsorption capacity of these chitosan-based adsorbents toward Cr(VI) ranged from 3.2 to 344.8 mg/g and the optimum pH mostly occurred at pH < 4.0 (Table 1). Cross-linked chitosan beads, which were synthesized by 1,2,7,8-diepoxyoctane (DEO) (as a crosslinker) and chitosan beads, exhibited a maximum adsorption capacity of 325.2 mg/g toward Cr(VI) at an initial concentration of 20–500 mg/L and pH 2.0 and 30 °C [10]. Meanwhile, chitosan composites could also overcome the shortage of chitosan alone. For example, the maximum adsorption capacity of graphene oxide/chitosan/ferrite nanocomposites toward Cr(VI) at concentration of 10–125 mg/L at pH 2.0 and 27 °C reached 270.3 mg/g [78], much higher than that of the chitosan alone. Whereas, the progressive hydrolysis of polysaccharide on chitosan during the regeneration by NaOH or acid treatments remains the roadblock of its application [24].

### 2.5. Clay-Based Adsorbents

**Figure 4:**
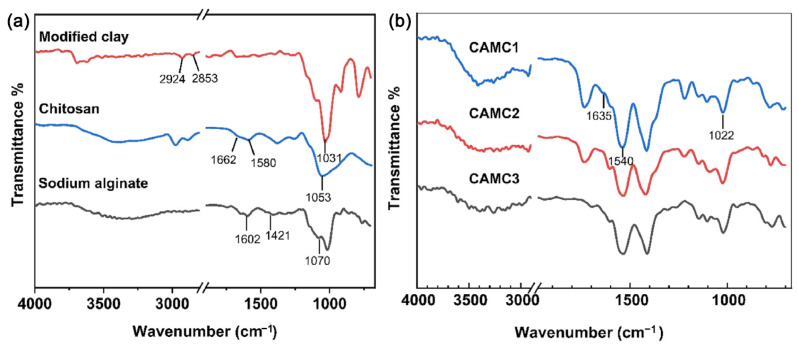
FTIR spectra of (**a**) modified clay, chitosan and sodium alginate and (**b**) chitosan/alginate/modified clay composite aerogel with the ratio of chitosan: sodium alginate: modified clay at 7:3:1.4 (CAMC1), 6:4:1.2 (CAMC2) and 5:5:1 (CAMC3). Reprinted with the permission from Ref. [87]. Copyright 2021 Elsevier.

Despite the application of clay-polymer absorbents for removing pollutants has been reviewed in several literatures [83], a review specific for Cr(VI) has not been reported. Endowed with high specific surface area, extraordinary cation exchange capacity (CEC) and cation exchange selectivity, surface hydrophilicity, and surface electronegativity, clay-based absorbents have drawn widespread attention for heavy metal removal [84]. However, the recovery/regeneration of these clays is hard and tedious, and they are sometimes ineffective for removing pollutants at trace levels [85]. The combination of polymers and clays can maximize the advantages of clays. Functional groups, such as hydroxyl (–OH), carboxyl (–COOH) and amine (–NH_2_) groups, could be easily introduced into the surface or interlayers of clays through polymerization [84]. FTIR spectra of the modified clay, chitosan sodium alginate, and chitosan/alginate/modified clay composite aerogel (CMAC) showed the presence of –OH, –COOH and –NH_2_ groups (Figure 4). Polymer modified clay can form a specific interaction with contaminants, resulting in strong adsorption or even selective adsorption [84]. Clays, including montmorillonite [86], kaolinites [87], calcium rectorite [88], sepiolite [26], and halloysite [89], have been utilized as fillers in polymers. According to Table 1, the maximum capacity of clay-based absorbents ranged from 42.4 to 714.3 mg/g, and the optimum pH values were mainly at 1.0–2.0. For example, the maximum adsorption capacity of PPy-coated halloysite nanotube nanocomposites toward Cr(VI) was calculated to be 149.3 mg/g at the concentration of 100–500 mg/L under pH 2.0 at 25 °C [90]. PPy coated sepiolite fiber retains the feature of sepiolite fiber templates but have a larger surface area [26]. Noteworthy, the prices varied among the clays and polymers. Thus, the synthesis of green and innovative clay–polymer composites based on low-cost and natural materials could be the direction of future efforts [91]. Moreover, the leaching of metals from metal-doped clay-polymer nanocomposites also deserves attention.

### 2.6. Nano-Sized Metals/Metal Oxides Based Polymeric Adsorbents

**Figure 5:**
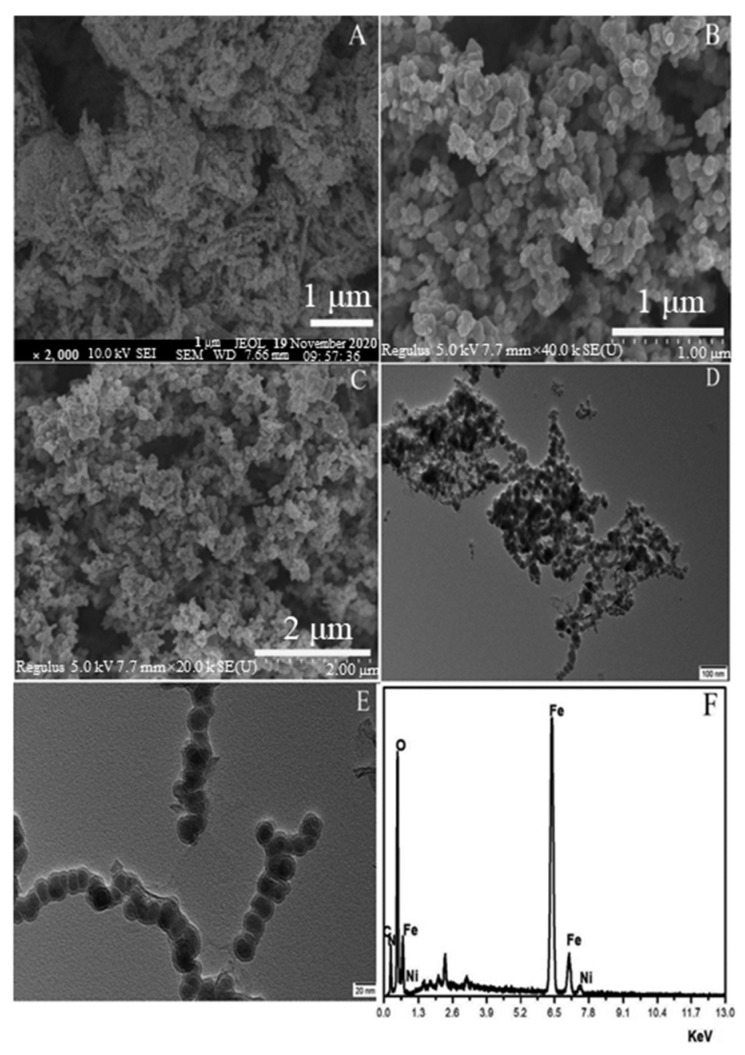
SEM image of nZVI (**A**); SEM images of PVP-nZVI/Ni (**B**,**C**); TEM images of PVP-nZVI/Ni (**D**,**E**); EDS of PVP-nZVI/Ni (**F**). Reprinted with the permission from Ref. [92]. Copyright 2022 Elsevier.

Metals/metal oxides based polymeric adsorbents for Cr(VI) have not been reviewed before. Nano-zerovalent iron (nZVI), which has a metallic iron core and iron oxide shell, has been applied for Cr(VI) removal [92]. The metallic iron core possesses the well-characterized electron-donating power, while the surface iron hydroxides offer the coordinative/electrostatic functions to attract and adsorb charged ions [92]. Both effects participate in the removal of metal ions. However, nZVI particles are susceptible to agglomeration and surface passivation to reduce their reactivities [93]. Coating of nZVI particles with polymers (e.g., sodium carboxymethyl cellulose [94], chitosan [95], and polyvinylpyrrolidone [92]) can increase the steric hindrance and electrostatic repulsion between the nanoparticles, thereby reducing the agglomeration of these nanoparticles. A polyvinylpyrrolidone stabilized iron-nickel bimetallic nanocomposite (PVP-nZVI/Ni), which was synthesized by sodium borohydride liquid phase reduction method, was effective for Cr(VI) removal at the pH values of 2.0–7.0 [92]. The maximum adsorption of PVP-nZVI/Ni toward Cr(VI) was calculated as 400 mg/g at concentrations of 0–10 mg/L under pH 2.0 at 25 °C [92]. As can be seen in Figure 5, pure nZVI exhibited an obvious chain-like aggregation structure, while PVP-nZVI/Ni after modification showed better dispersion. Additionally, these nano-sized metals based absorbents were also robust for Cr(VI) removal at relatively low Cr(VI) concentrations (Table 1). Nano-sized metal oxides, such as thorium oxide [96], titanium oxide [97], zinc oxide [98], manganese oxide [99], and ferric oxide [100], have also been used for Cr(VI) removal due to their vast surface area, high capacity and selectivity. However, the treatment performances of these sole metal oxides are far from ideal. For example, the highest adsorption capacity of alumina toward Cr(VI) was only 52.1 mg/g [101]. Modifying of metal oxides with polymeric materials improved the Cr(VI) removal. The maximum sorption capacity of xanthated chitosan-wrapped γ-Al_2_O_3_ composite toward Cr(VI) reached 110.5 mg/g at concentrations of 10–80 mg/L under pH 3.0 and 25 °C [102]. As shown in Table 1, the BET surface areas of nano-sized metals or metal oxides based absorbents are relatively higher than those of other absorbents, which are in the range of 16.3–272.5 m^2^/g. The maximum sorption capacities of nano-sized metals or metal oxide based absorbents were in the range of 13.5–549.4 mg/g and the optimum pH often occurred at pH 2.0 (Table 1). For instance, a polyaniline-coated tungsten trioxide biphasic composite showed maximum adsorption capacity of 549.4 mg/g toward Cr(VI) ranging from 0–150 mg/L at pH 2.0 [103]. However, the research on nano-sized metals/metal oxides based polymeric adsorbents is still an emerging field, and more efforts are needed to explore the potential of extensive use of these novel adsorbents in practical production.

### 2.7. Carbon-Based Absorbents

The removal of pollutants using carbon-based absorbents has been intensively reviewed in the literatures, while how these novel materials could affect the Cr(VI) has rarely been studied [83]. Carbon materials with high specific surface areas and hierarchically porous networks are efficient adsorbents for heavy metals [104]. These carbons include activated carbon (AC), graphene oxide (GO), biochar, carbon nanocomposites, carbon nanotubes, carbon sponges, carbon aerogels, and carbon membranes [104]. Theoretically, all of the organic materials can act as the precursor of AC. The porous structure and surface properties of AC determined the sorptive removal of Cr(VI) [105]. GO has large amounts of oxygenated functional groups arranged in sp2-hybridized shape, which forms a honeycomb structure [106]. This unique structure of GO promotes the formation of a network with Cr(VI) through covalent and ionic bonds [106]. Biochar has a high specific area and abundant functional groups on its surface, which exhibits good performance in treating Cr(VI) [107]. However, the reuse of AC is hard [108], the agglomeration of GO is serious [109], and the surface of biochar is typically negatively charged to resist the adsorption of anions [3]. These adverse effects hinder Cr(VI) removal by carbon materials. The combination of carbon materials and polymers provides a solution to improve the Cr(VI) removal. Coating of polysulfide rubber on activated carbon increased the Cr(VI) removal by 8.0% [108]. GO modified by poly(allylamine hydrochloride) cross-linked amino exhibited a maximum adsorption capacity of 373.1 mg/g for Cr(VI) at the concentrations of 10–780 mg/L at pH 2.0 and room temperature, which was ~9 times higher than that of pure GO [4]. What’ more, it also performed well in eliminating Cr(VI) at low concentrations, which reduced Cr(VI) from 9.9 to 0.004 mg/L within 10 s with the dosing of 50 mg modified GO [4]. Yang et al. also reported that magnetic corncob (MBC/PPy) performed well to removing Cr(VI) at low concentrations [30]. The maximum adsorption capacity of MBC/PPy was calculated to be 19.23 mg/g at concentrations of 20–60 mg/L at pH 5.3 and 20 °C [30]. Furthermore, MBC/PPy was proven to reduce Cr(VI) [30]. Cr(VI) and Cr(III) accounted for 20.6% and 79.4% of the total absorbed Cr, respectively [30]. Table 1 shows the performance of carbon-based adsorbents. The BET surface areas of carbon-based material absorbents are 11.2–688.6 m^2^/g (Table 1). The maximum adsorption capacities of these carbon-based adsorbents toward Cr(VI) ranges from 8.9 to 1185.0 mg/g (Table 1). The initial Cr(VI) concentrations investigated are in the range of 2.0–2500 mg/L (Table 1). Thus, carbon-based materials absorbents show great potential to eliminate Cr(VI) in wastewater with various initial concentrations. However, the overall costs of these carbon-based polymers are high, and the recovery efficiencies and selectivity for Cr(VI) are low. Thus, more researches are imperative to reduce the treatment cost and to enhance the reusability of these materials to address the selective adsorption of Cr(VI) under complicated conditions.

**Table 1:** Summary of removal of Cr(VI) using polymeric adsorbents.

Categories	Adsorbents	pH	T (°C)	q_max_ (mg/g) ^a^	C_0_ (mg/L) ^b^	Removal Rate (%)	Driving Force for Cr(VI) Removal	pH_pzc_^c^	S_BET_ (m^2^/g) ^d^	Main Groups	Advantages	Disadvantages
Polypyrrole-based adsorbent	Polypyrrole–coated gum ghatti–grafted poly(acrylamide) composite [17]	2.0	25	321.5	25–100	99.6	--	--	15.9	–CONH_2_	Unique redox chemistry; nontoxicity; environmental stability.	Low surface area and adsorption capacities; costly or complex synthesis of composites in most cases; difficult and time consuming recovering and settling.
	Graphene/SiO_2_@polypyrrole nanocomposites [18]	2.0	25	429.2	60–100	--	Electrostatic attraction; ion exchange; chelation; reduction	--	37.6	–COOH; –OH; –NH_2_		
	Free standing polypyrrole film and powder [28]	3.0	30	103.6	50–200	69.5	Electrostatic attraction; ion exchange	--	--	–Cl; –NH_2_; –NH–		
	Polypyrrole/m–phenylediamine/Fe_3_O_4_ nanocomposite [29]	2.0	25	555.6	100–600	100.0	--	2.3	120.6	–NH_2_		
	Three–dimensional flowerlike nanospheres composed of molybdenum disulphide and polypyrrole [31]	2.0	25	231.0	50	100.0	Adsorption; reduction	3.8	149.8	–NH–; –NH=		
	Polypyrrole –iron oxide–seaweed nanocomposite [100]	2.0	30	144.9	50–250	96.4	Electrostatic interaction; ionexchange; reduction	3.8	--	–Cl; –NH_2_; –COOH; –OH		
	Poly(pyrrole)–based magnetic hydrogel nanocomposite [110]	1.0	65	208.0	50–150	--	--	6.0	--	–COOH; C=O; –NH–		
	Sulfonated poly(arylene ether nitrile)/polypyrrole core/shell nanofibrous mat [111]	2.0	25	165.3	25–200	56.5	Electrostatic attraction	--	--	–NH–; –N=		
	Bamboo–like polypyrrole nanofibrous mats [112]	2.0	25	961.5	50–250	65.0	Electrostatic attraction; anion exchange; reduction	--	37.4	–Cl; –NH_2_		
	Poly(1H–Pyrrole–1–ethanamine) [113]	1.6	25	729.1	20–300	99.0	Electrostatic interaction; reduction; hydrogen bonding	10.2	50.2	–NH_2_		
Polyanline–based composites	Copper ferrite–Polyaniline nanocomposite [44]	2.0	25	1000.0	20–60	97.0	--	–	--	–OH; –NH_2_	High conductivity; simple preparation; reasonable stability; low–cost; biocompatible.	Low adsorption capacity; largely depends on the material nature; the close control of pH and temperature requirements during production process.
	Polyaniline confined in pores of polystyrene beads [48]	6.0	25	233.7	10–200	100.0	Electrostatic attraction; reduction; chelation	--	652.6	–NH–; –N=		
	Hollow spherical polyaniline [50]	1.0	25	601.3	40–180	100	Reduction; chelation; electrostatic interactions	--	14.1	–NH–; –N=		
	Porous polyaniline synthesized with the bacteria cell [51]	1.0	25	835.1	1–1500	99.0	Electrostatic interactions; reduction	4.2	51.2	–NH–; –N=		
	Fe_3_O_4_@polyaniline/itaconic acid magnetic nanocomposite [55]	2.0	--	218.0	10–1000	92.2	Electrostatic interactions	--	--	–NH_2_; –COOH; –OH		
	Polyaniline coated bacterial cellulose mat [56]	7.0	25	--	50–800	100.0	Adsorption; reduction	7.2	--	–OH; –NH_2_		
	Bacterial cellulose/polyaniline nanocomposite aerogels [62]	7.0	25	189.0	50–300	68	--	5.5	58.0	–OH; –NH_2_		
	Carboxymethyl cellulose/polyaniline nanocomposite [114]	2.0	25	137.0	10–200	89.4	--	4.2	3.0	C–O–C; –COO^−^		
	Lignin/polyaniline composites [115]	4.0	--	25.0	50	>99.0	Electrostatic interaction; reduction	--	--	–NH_2_		
	Para toluene sulfonic acid immobilized–polyaniline@carbon nanotubes nanocomposites [116]	2.0	30	166.7	25–200	--	Electrostatic interactions; reduction	--	--	–NH–; –N=		
	Polyaniline@Ni(OH)_2_ nanocomposites [117]	4.0	25	625.0	50–150	100.0	Complexation; reduction	–	26.9	–OH; –NH–; –N=		
	Arginine–functionalized polyaniline@FeOOH composite [118]	2.0	20	682.3	100–700	98.1	Electrostatic interaction; reduction; complexation	4.0	--	–OH; –NH–; –NH_2;_ –N=		
Cellulose–based adsorbent	Hierarchical polydopamine coated cellulose nanocrystal microstructures [11]	3.0	15	205.0	30–600	>90.0	Adsorption; reduction	--	101.9	–OH; –NH_2_; –N=; C=O	Wide availability; non-toxicity; good chemical stability; excellent mechanical properties; high hydrophilicity; presence of large number of functional groups; flexible structure of the polymer chain.	Time–consuming; poor regeneration performance; complicated separation procedures from aqueous solution; low removal rate at low Cr(VI) concentration
	Cellulose nanofibrils grafted with poly(m–aminobenzene sulfonate) [65]	1.0	25	5.3	1–60	100.0	Electrostatic interaction; reduction	--	--	–COOH; –NH–; –OH; C=O; –NH–; C–S		
	Polyaniline–impregnated nanocellulose composite [67]	6.0	--	92.6	10–500	96.5	Electrostatic interaction; ion exchange; reduction; complexation	--	--	–NH–; –NH_2_; –OH		
	Cellulose –based adsorbent with functional groups of quaternary ammonium and amino [69]	2.0	30	490.3	20–700	99.0	Electrostatic adsorption; reduction	9.6	--	–OH; –NH_2_; ^+^N≡		
	Acrylamidethiosemicarbazide cellulose aerogels [70]	3.0	25	83.4	1–20	100.0	Electrostatic attraction; reduction; chelation	5.1	19.4	–OH; C–S; C=S; –NH_2_		
	Carboxymethyl cellulose/polyethylenimine hydrogel [119]	3.0	25	312.5	52–520	--	Electrostatic adsorption; reduction; coordination; precipitation	9.0	--	–OH; –COOH; –NH–; –NH_2_		
	Polyethylenimine–grafted magnetic cellulose [120]	3	30	198.8	0–120	100.0	Electrostatic adsorption; hydrogen binding; chelation	--	--	–NH_2_		
	Cellulose–based solid amine adsorbent with high amino density [121]	4.0	30	327.7	0–550	100.0	Electrostatic interactions; chelation; redox	--	--	–OH; –NH_2_–		
Chitosan–based adsorbent	Tetraethylenepentamine crosslinked chitosan oligosaccharide hydrogel [16]	3.0	30	148.1	0.02–800	100%	Electrostatic interaction; coordination	8.5	--	–OH; –NH_2_; –NH–	Low–cost; biodegradability; high sensitivity to a variety of contaminants; high reactivity; presence of positions for modification in its chemical structure.	High crystallinity; low mechanical strength; instability in acidic medium; low specific area and porosity in powder and flake forms; low regeneration ability.
	Graphene oxide/chitosan/ferrite nanocomposite [78]	2.0	27	270.3	10–125	96.0	--	8.0	74.4	–OH; C=O; C–O–C		
	Lignocellulosic derivative and chitosan bioadsorbent [80]	2.5	20	115.8	25–450	92.8	--	--	--	–OH; –NH_2_; C=O; C–O–C		
	Heterocyclic modification of chitosan [81]	3.0	--	85.0	20–100	--	Electrostatic interaction	--	--	–OH; –Cl; –NH–; –NH=; C=O		
	Magnetic chitosan chloride–graphene oxide–metal oxide composites [99]	2.0	25	78.2	1–100	--	Electrostatic interactions; cation exchange	--	--	–OH; –Cl; C=O; –N≡		
	Chitosan–stabilized FeS magnetic composites [122]	3.0	20	119.3	25–100	100	Adsorption; reduction; complexation	4.7	14.1	–OH; –NH_2_; Fe–O; –S		
	Chitosan beads modified with sodium dodecyl sulfate [123]	4.0	25	3.2	1	--	--	--	--	–OH; –NH_2_; C–O–S; C=O		
	Poly(4–vinyl pyridine) decorated magnetic chitosan biopolymer [124]	2.0	25	344.8	100–700	--	Electrostatic interaction; reduction; coordination	7.7	0.6	–N=; –NH_2_		
	Chitosan and corn stover derivative bioadsorbent [125]	3.0	25	80.3	250	--	--	--	--	–OH; –NH_2_		
	(Chitosan–g–PMMA)/Silica Bionanocomposite [126]	4.0	25	92.5	10–500	--	--	--	--	–OH; –NH_2_; C=O; C–O		
	Sponge–like kaolin/chitosan/reduced graphene oxide composite [127]	1.0	35	43.1	10–170	>94.0	Electrostatic interaction; π-π stacking interaction; the subsidiarity of π electrons carbocyclic sixmembered ring; reduction	--	6.1	–OH; –NH_2_; –COOH; C=O; –NHCO–		
	Zirconium impregnated chitosan microspheres [128]	4.5	25	230.4	0–150	--	Electrostatic interaction; complexation	--	--	–OH; –NH–		
Clay–based adsorbents	Polypyrrole coated sepiolite fiber [26]	2.0	25	108.9	20–100	-- ^e^	Electrostatic attraction; anion exchange; reduction	6.2	41.0	–Cl; –N=	High removal efficiency; low cost; high efficiency in small amounts; ease to apply at small scale.	Low degradation and mobility; harmful high metal concentration in metal–doped clay–polymer nanocomposites.
	Chitosan/alginate/modified clay composite aerogel [87]	3.1	25	62.4	50–400	78.2	Electrostatic interaction	--	--	–OH; –COOH; –NH_2;_ C=O		
	Polypyrrole/calcium rectorite composite [88]	1.5	25	714.3	50–200	100.0	Electrostatic interactions; reduction; ion exchange	11.0	--	–Cl; –OH; –NH–; –COOH		
	L–cysteine doped polypyrrole/bentonite composite [129]	1.0	25	318.5	50–700	--	Electrostatic attraction; ion exchange; chelation; reduction	3.0	--	–COOH; –NH_2_; –NH–; –NH=		
	Polypyrrole–montmorillonite clay composite [130]	2.0	ambient temperature	--	100	100.0	Electrostatic attraction; ion exchange; reduction	--	--	C–N		
	Cetylpyridinium chloride–[Al_30_O_8_(OH)_56_(H_2_O)_24_]^18+^ pillared montmorillonite composite [131]	2.0	25	42.4	0–1000	92.38	Electrostatic interaction; ion exchange; reduction	7.0	18.3	–Cl; –OH; C=N		
Nano–sized metals or metal oxides based adsorbents	Poly(catechol–1,4–butanediamine)–coated Fe_3_O_4_ composite [12]	2.5	20	280.1	0–200	100.0	Adsorption; reduction	--	--	–OH; –NH–	High thermal stability; readily availability; chemica stability over a wide range of pH; ability to bind to cationic or anionic ligands selectively; nontoxicity; low cost; high surface areas; large number of active sites.	Low adsorption capacity.
	Nanostructured iron oxide stabilized by chitosan [14]	3.0	25	112.0	2.5–750	73.6	Electrostatic interaction; reduction; strong interactions	--	--	–OH; –NH–; –NH_2_		
	Neodymium–doped polyaniline supported Zn–Al layered double hydroxide nanocomposite [20]	--	25	219.0	0.4–454	>97.0	Electrostatic interaction	5.5	--	–OH; –NH_2_; –NH=; –NH–		
	Polyvinylpyrrolidone supported nzvi/Ni bimetallic nanoparticles [92]	2.0	25	385.4	10–50	>95.0	Adsorption; reduction; coprecipitation	--	272.5	–OH; C–N		
	Modified thorium oxide polyaniline core–shell nanocomposite [96]	5.0	30	141.0	10–100	>85.0	Electrostatic interactions; reduction	--	87.0	–NH_2_; –NH–; –NH=		
	Polyaniline–coated tungsten trioxide biphasic composite [103]	2.0	25	549.4	20–500	95.1	Electrostatic attraction; reduction; transfer of mass phenomenon	3.6	16.3	–NH=; –NH–; –NH_2_		
	Fe_3_O_4_ anchored polyaniline intercalated graphene oxide [106]	3.0	ambient temperature	143.5	50–200	--	Electrostatic attraction; complexation	--	58.0	–NH=; –NH–; –NH_2_; C=O; –COOH		
	Hydrous ferric oxide nanoparticles hosted porous polyethersulfone adsorptive membrane [132]	2.0	--	13.5	5–50	>75.0	--	--	233.7	–OH; C–O–C; O=C=O		
	Porous polyaniline/itaconic acid–based CuO nanocomposite material [133]	2.0	25	250.0	10–1000	96.0	--	--	--	–OH; C=O, C–O–H; C–O; C–N^+^		
	Micrometer–sized polystyrene/polyaniline–Fe_3_O_4_ composite [134]	2.0	25	23.8	10–40	100.0	Ion exchange	--	--	–NH–		
	Calcined Mg/Al–layered double hydroxides/polyaniline composites [135]	2.0	20	409.8	10–120	--	Electrostatic attraction; reduction; complexation; chelation	6.3	49.2	–Cl; –NH–; –NH=		
	Magnetic polypyrrole–polyaniline/Fe_3_O_4_ nanocomposite [136]	2.0	25	303.0	100–600	99.0	Ion exchange; reduction	--	56.5	–NH=; –NH–; –NH_2_; –Cl		
Carbon–based adsorbents	Poly(allylamine hydrochloride) covalently cross–linked amino–modified graphene oxide [4]	2.0	25	373.1	9.9–856.4	100.0	Electrostatic attraction; reduction; chelation	--	261.6	–OH; –COOH; –NH_2_	Large porous surface area; significant stability; tunability of the surface and structure; high degree of surface reactivity.	High cost; no selective properties for chromium adsorption; low recovery efficiency.
	Magnetic corncob biochar/polypyrrole composite [30]	5.3	20	19.2	10–30	--	Ion exchange; reduction; precipitation; chelation	7.0	--	–Cl; –OH; –NH=; C=O; C–O		
	Activated carbon modified by polysulfide rubber coating [108]	4.0	22 ± 1.0	9.0	2–20	98.0	Electrostatic attraction; reduction	4.8	688.6	–Cl; C–S; S–H		
	Polyethylenimine grafted amino–functionalized graphene oxide nanosheets [109]	2.0	25	1185.0	20–2500	100.0	Electrostatic attraction; reduction	--	--	–OH; –NH=; –NH–; –NH_2_; C=O		
	Polymer–mediated nitrogen–rich reduced graphene oxide [137]	2.0	25	417.0	10–250	99.0	Electrostatic attraction; reduction; chelation	8.2	--	–OH; –NH_2_		
	Polyacrylonitrile/Pterocladia capillacea–activated carbon [138]	1.0	25	344.8	50–250	100.0	Electrostatic interactions	5.7	11.2	–OH; –NH_2_		

Notes: q_max_ (mg/g) ^a^: Maximum adsorption capacity; C_0_ (mg/L) ^b^: Initial Cr(VI) concentrations; pHpzc ^c^: Isoelectric point; S_BET_ (m^2^/g) ^d^: Brunauer–Emmett–Teller surface area; -- ^e^: Not reported.

## 3. Mechanisms of Cr(VI) Removal

**Figure 6:**
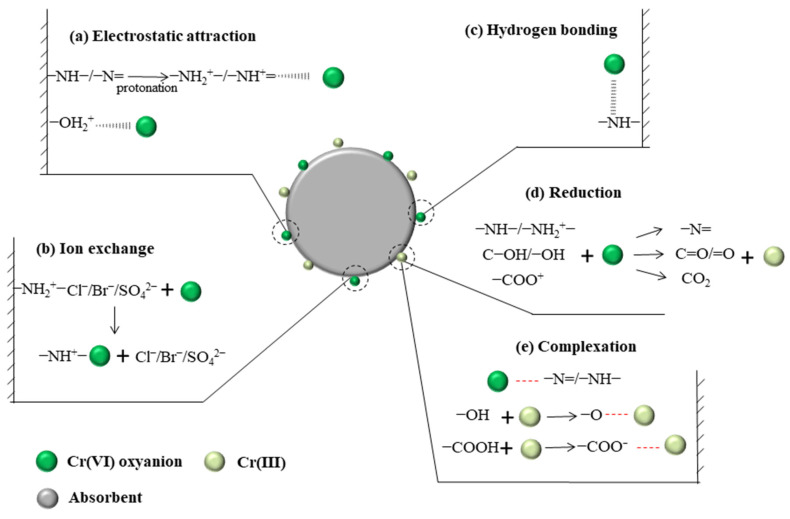
Mechanistic schematic diagram for Cr(VI) removal by polymeric adsorbents.

**Figure 7:**
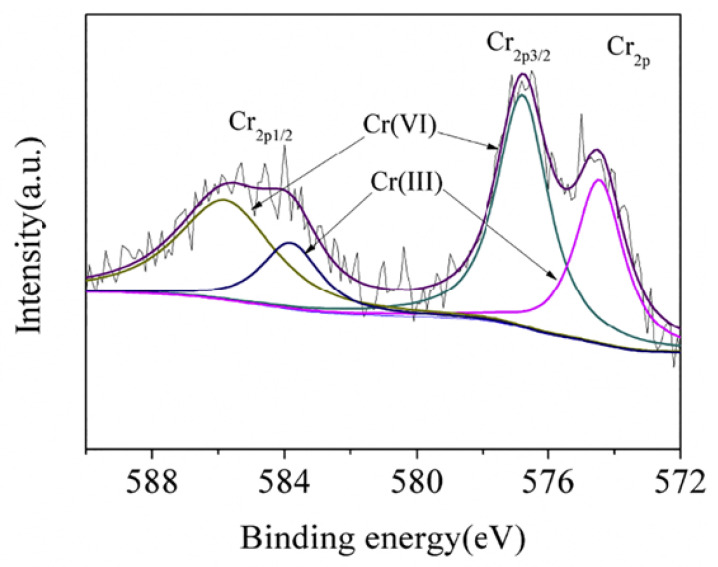
Cr_2p_ XPS spectrum of terminal amino hyperbranched polymer modified graphene oxide adsorbents (GO-HBP-NH_2_-TEPA) after the adsorption of Cr(VI). Reprinted with the permission of Ref. [145]. Copyright 2019 Elsevier.

**Figure 8:**
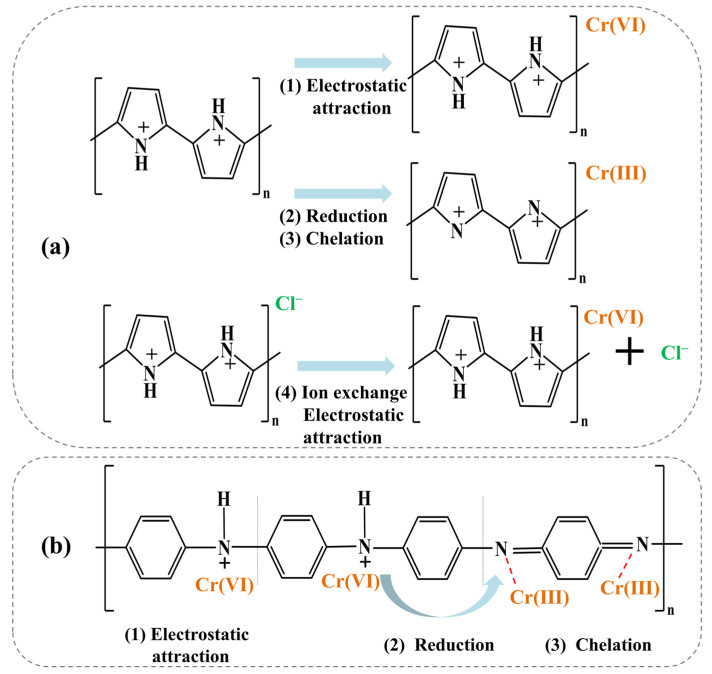
Adsorption of Chromium ions on the (**a**) polypyrrole and (**b**) polyaniline.

**Figure 9:**
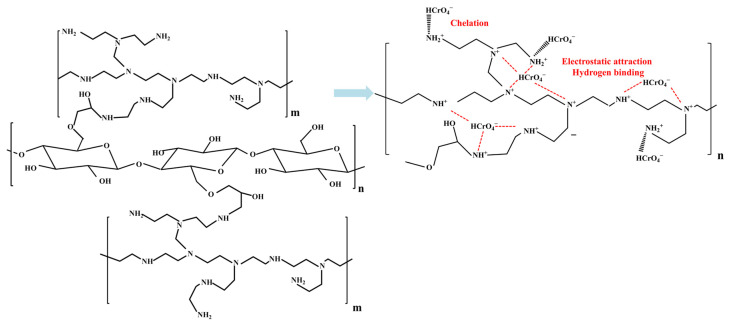
Mechanistic schematic diagram of Cr(VI) adsorption on polyethyleneimine-grafted magnetic cellulose. Reproduced from Ref. [120].

The driving force of Cr(VI) removal varies by the properties of the adsorbents and the working conditions. Techniques, including Fourier transform infrared spectroscopy, X-ray photoelectron spectroscopy, and X-ray absorption near edge structure spectroscopy are generally used to elucidate the mechanism of Cr(VI) removal [7,139]. Mechanisms of electrostatic attraction, anion exchange, chelation, complexation, and hydrogen bonding are involved in Cr(VI) removal (Figure 6). Different functional groups play different roles in Cr(VI) removal. As shown in Table 1, nitrogen-containing groups in polymers exhibit a pronounced effect on Cr(VI) removal. –NH– and –N= groups are easy to protonate to –NH_2_^+^– and –NH^+^= in acidic medium, respectively [51]. Electrostatic attractions between these positively charged sites and Cr(VI) anions (i.e., HCrO_4_^−^ and Cr_2_O_7_^2−^) could enhance Cr(VI) removal [140]. Oxidation of –NH– to =N– by Cr(VI) generates Cr(III) [141], which has strong affinity toward amine groups [142]. Besides, these nitrogen-containing groups could also improve the hydrophilicity and the charge density of the polymer surfaces, which are helpful for Cr(VI) removal [137]. The doped nitrogen on polymers creates more basic sites which are capable of providing more delocalized electrons to enhance the reduction of Cr(VI) [137]. Meanwhile, the doped-N also results in charge redistribution, which may subsequently induce the adjacent C atoms to exhibit the feature of Lewis bases to bind Cr(III) ions (Cr^3+^ and Cr(OH)^2+^) [137]. Furthermore, nitrogen atoms have a strong ability to accept electrons, and the carbon atoms adjacent to them generally carry net positive charges, which contribute to the Cr(VI) removal through electrostatic attraction [137]. Except that, hydrogen bond force, coordination interaction, cation exchange and chelating interaction, are also involved in the adsorption of Cr(VI) introduced by nitrogen-containing groups [64,143]. Because of the presence of electron-donating groups (e.g., amine/imine groups, carboxyl, hydroxyl, and phenol) in polymeric materials (Table 1), the reduction of Cr(VI) to Cr(III) often occurs during the reactions between these adsorbents and Cr(VI). Figure 7 exhibited the XPS spectrum of terminal amino hyper branched polymer modified graphene oxide adsorbents (GO-HBP-NH_2_-TEPA) after the adsorption of Cr(VI). The presence of characteristic peaks of Cr(III) (~34.6%) confirmed the reduction of Cr(VI) by amine groups on the GO-HBP-NH_2_-TEPA (Figure 7). Carboxyl and hydroxyl groups are effective for Cr(III) removal through complexation or chelation mechanisms [30,141]. The doping of anions into the structure of polymers can enhance Cr(VI) via anionic exchange mechanism, and Cl^−^ and SO_4_^2−^ are the two most common doping anions [18,144]. For the specific types of adsorbents, the main driving force of Cr(VI) removal are varied with each other. Electrostatic attraction and anion exchange are the main driving force of Cr(VI) removal by PPy based adsorbents due to the presence of amino groups and Cl^−^ in their structures (Figure 8). The removal of Cr(VI) by PANI-based composites is mainly attributed to the electrostatic interaction and reduction caused by the presence of imine and amino groups (Figure 8). It is worth noting that hydroxyl groups on the cellulose- and chitosan-based absorbents play a significant role in the chromium removal through chelation. At the meantime, the imine and amino groups also contribute to the adsorption of Cr(VI) on the cellulose- and chitosan-based absorbents through electrostatic interaction (Table 1). Figure 9 shows the Cr(VI) adsorption through electrostatic attraction, hydrogen binding and chelation with amino groups on polyethyleneimine-grafted magnetic cellulose. Electrostatic attraction, ion exchange, chelation, and reduction all work during the treatment of Cr(VI) by clay-based adsorbents (Table 1), while the main driving force is hard to be identified. The hydroxyl, quinoid imine, imine and amino groups facilitate nano-sized metals or metal oxides- and carbon-based materials absorbents to immobilize Cr(VI) by electrostatic interactions and reduction (Table 1). These mechanisms often work together to realize the efficient Cr(VI) removal, and they are hard to be quantified. In spite of this, an in-depth investigation of the mechanism for Cr(VI) removal becomes even more important on designing more robust adsorbents.

## 4. Factors Affecting Cr(VI) Removal

### 4.1. Solution pH

**Figure 10:**
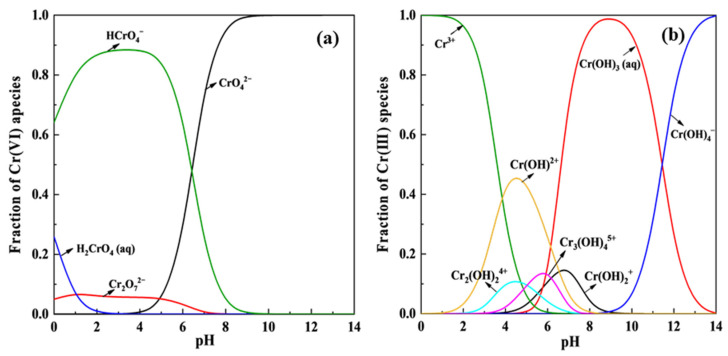
Chemical speciation of (**a**) Cr(VI) and (**b**) Cr(III) at the solution pH of 0–14.0.

**Figure 11:**
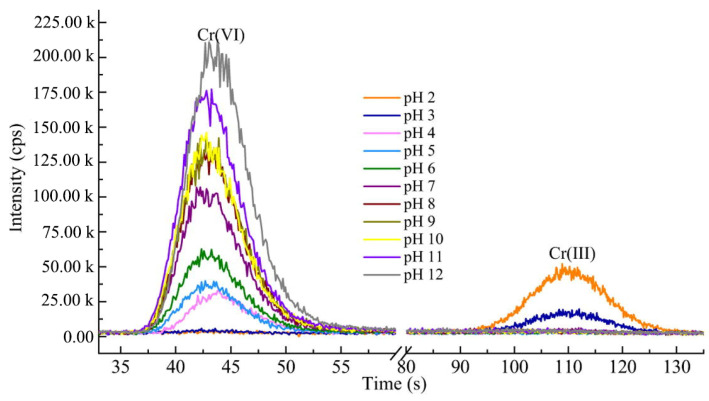
Cr speciation in solutions obtained by IC–ICP–MS chromatograms during the reactions between Cr(VI) and magnetic PPy–PANI/Fe_3_O_4_ nanocomposite. Reproduced with the permission from Ref. [136]. Copyright 2017 Elsevier.

Solution pH, which affects both the surface characteristics of the adsorbent and the forms of Cr(VI) [146], is a critical factor determining the adsorption process. Generally, the removal of Cr(VI) by polymeric absorbents decreases with the increase of pH [147]. The maximum adsorption mostly occurred at pH values of 2.0–4.0 (Table 1). As shown in Figure 10, the speciation of Cr(VI) shifts from H_2_CrO_4_ to HCrO_4_^−^ and CrO_4_^2−^ as the solution pH increases from 1.0 to higher than 6.0. Meanwhile, the redox potential of Cr(VI) decreases with its deprotonation, making Cr(VI) easier to be reduced to Cr(III) in acidic medium [39]. Kera et al. analyzed the concentrations of Cr(VI) and Cr(III) in solutions during the sorptive removal of Cr(VI) using PPy-PANI/Fe_3_O_4_ nanocomposite at the initial concentrations of 2.0–12.0 (Figure 11) [136]. The presence of Cr(III) and the absence of Cr(VI) at initial pH values of 2.0 and 3.0 confirmed the reduction of Cr(VI) to Cr(III) by the PPy-PANI/Fe_3_O_4_ nanocomposite (Figure 11). –NH_2_ and –OH are easy to protonate to –NH_3_^+^ and –OH_2_^+^, respectively, which facilitate the sportive removal of Cr(VI) anions via electrostatic attraction and hydrogen bonding [148]. The positive charges on adsorbent surfaces increase with the decrease of pH, which is favorable for the adsorption of anionic Cr(VI) species through electrostatic attraction [149]. Sorbents with higher pHpzc carry more positive charges than those of with lower pHpzc. Moreover, OH^−^ may compete with Cr(VI) for the adsorption sites to reduce Cr(VI) removal [150]. To broaden the pH range of Cr(VI) removal, the synthesis of sorbents with high pHpzc could be a direction.

### 4.2. Initial Cr(VI) Concentration

**Figure 12:**
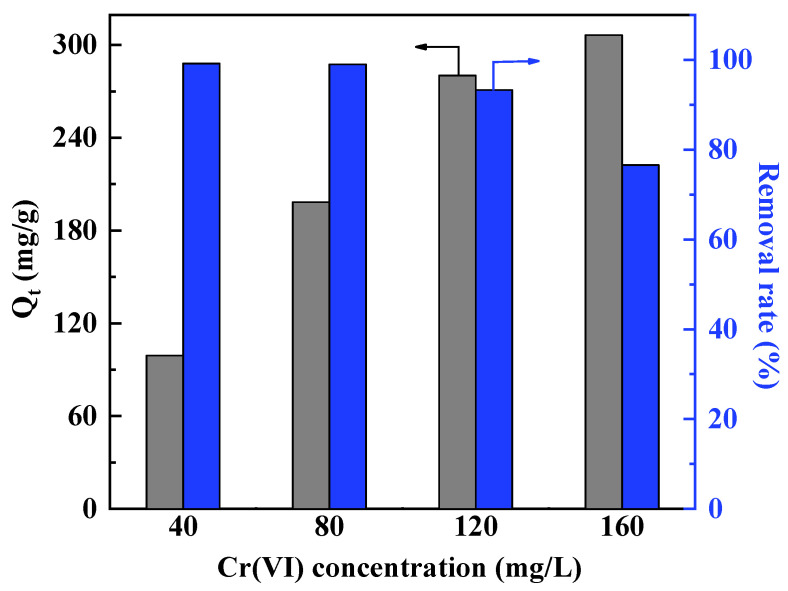
The effects of initial Cr(VI) concentration on the removal efficiency and adsorption capacity of UiO-66 derived N-doped carbon nanoparticles coated by PANI toward Cr(VI). Reproduced with the permission from Ref. [39]. Copyright 2019 Elsevier.

The maximum adsorption capacities of adsorbents increase with the initial concentration of Cr(VI), while the actual Cr(VI) removal efficiencies decreases with its concentration [39]. For example, the adsorption capacity of UiO-66 derived N-doped carbon nanoparticles coated by PANI toward Cr(VI) increased from 99.2 to 306.4 mg/g as the initial Cr(VI) concentration increased from 40 to 60 mg/L, while the removal efficiency of Cr(VI) decreased from 99.2% to 76.6% (Figure 12). Increasing the Cr(VI) concentration could enhance the mass transfer between the liquid and solid phases to maximize the utilization of the active sites on polymers [151]. However, because of the limited available active sites on the absorbent, the percent of Cr(VI) removed could be higher in the system with lower initial Cr(VI) concentration. Therefore, adsorbents, which are suitable for the removal of Cr(VI) at low concentrations needed to be further explored.

### 4.3. Dosage of the Adsorbents

**Figure 13:**
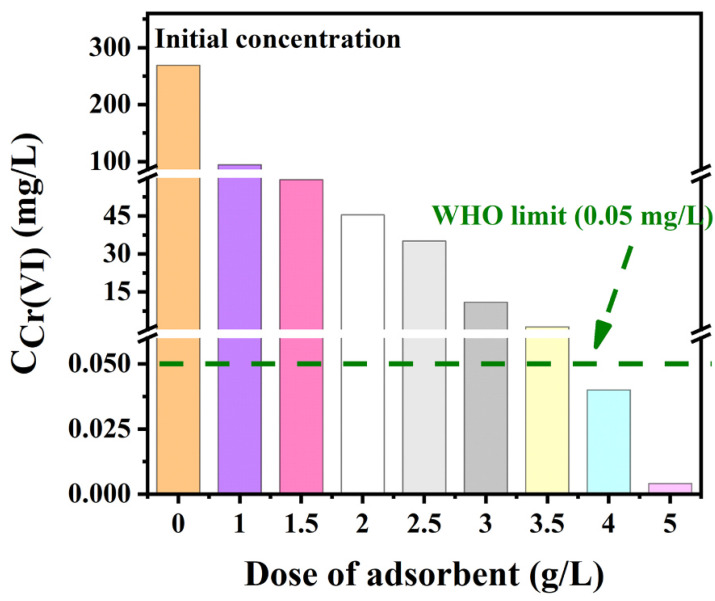
The effects of the adsorbent (poly(allylamine hydrochloride) covalently cross-linked amino-modified graphene oxide) dose on Cr(VI) removal. Reprinted with permission from Ref [4]. Copyright 2021 Elsevier.

There is no doubt that Cr(VI) removal increases with the rise of the adsorbents dosage [65]. A higher dosage of the adsorbents can provide more active sites to react and adsorb Cr(VI) [138]. As shown in Figure 13, as the dose of absorbent increased from 0 to 5 g/L, the residual concentration of Cr(VI) significantly diminished from 271.2 to below 0.05 mg/L. However, increasing the adsorbent dose to a certain extent could also result in the agglomeration to reduce adsorption [138]. Moreover, the high amount of adsorbent used could make the recycling of adsorbents difficult and thus increasing the overall treatment cost. Taken together, the dosage of adsorbent should be optimized to maximize the adsorption of Cr(VI) and to minimize the overall treatment cost.

### 4.4. Reaction Temperature

The adsorption of Cr(VI) is an endothermic reaction [100]. Increasing the reaction temperature can enhance the adsorption of Cr(VI), which can be explained by the following aspects. Firstly, rising of the temperature can increase the collision probability of Cr(VI) to react with the active sites. Secondly, increasing the temperature may decrease the viscosity of the solution to enhance the diffusion of Cr(VI) [50]. However, the high temperature could also result in the damage to active binding sites, weakening of binding forces, and a high cost of operation [100]. Thus, the adsorption of Cr(VI) typically operates at normal temperature.

### 4.5. Co-Existing Ions

**Figure 14:**
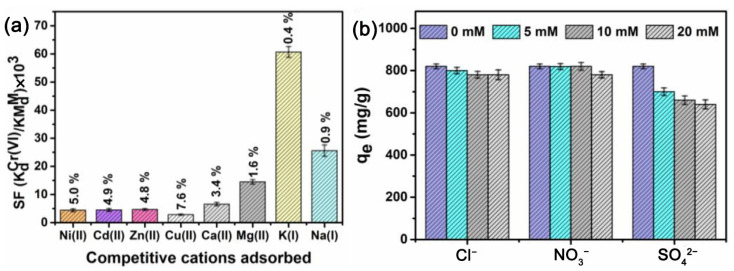
(**a**) The separation factor of coexistence cations and removal efficiency of cations in the mixture containing 100 mg/g coexisting ions, (**b**) The adsorption capacity of PEI grafted amino-functionalized graphene oxide nanosheets toward Cr(VI) with co-existing anions. Reprinted with the permission of Ref. [109]. Copyright 2020 Elsevier.

Cr(VI) coexists with various types of metal ions and anions in wastewater. The selectivity of the polymeric adsorbents is the prerequisite to realize efficient Cr(VI) removal in a complex matrix [73]. Because of the electrostatic repulsion between the protonated adsorbent and cations in acidic solution, cations barely affect the adsorption of Cr(VI) [109]. Meanwhile, H^+^ could also contend with the cations to minimize their interference on Cr(VI) adsorption [31]. However, the presence of anions (especially at high levels) can compete the adsorption sites to inhibit the Cr(VI) removal [102]. Meanwhile, the electrostatic repulsion between the Cr(VI) anions and the co-existing anions could be the another reason for inhibition [122]. Moreover, the thickness of the electrically diffused double layer between Cr(VI)-oxyanions and absorbents increased with the presence of coexisting anions, which could weaken the electrostatic attraction between Cr(VI) and the sorbent [146]. The inhibition effect increases with the charge carried by the anions [152]. The influence of co-existing ions on Cr(VI) adsorption can be assessed by the separation factor (SF). As shown in Figure 14, the SF was as high as 10^3^ − 10^4^, suggesting the high selectivity of PEI grafted amino-functionalized graphene oxide nanosheets (GONN) toward Cr(VI) in the presence of coexistent cations. SO_4_^2−^ showed a higher influence on the adsorption of Cr(VI) on the GONN than Cl^−^ and NO_3_^−^ because of the higher affinity of the amino ligands on GONN for SO_4_^2−^ [109]. Grafting and ion-templating are useful methods to increase the selectivity and feasibility of adsorbents for Cr(VI) in multi-ions system [153], and more efforts are needed.

### 4.6. Other Factors

**Figure 15:**
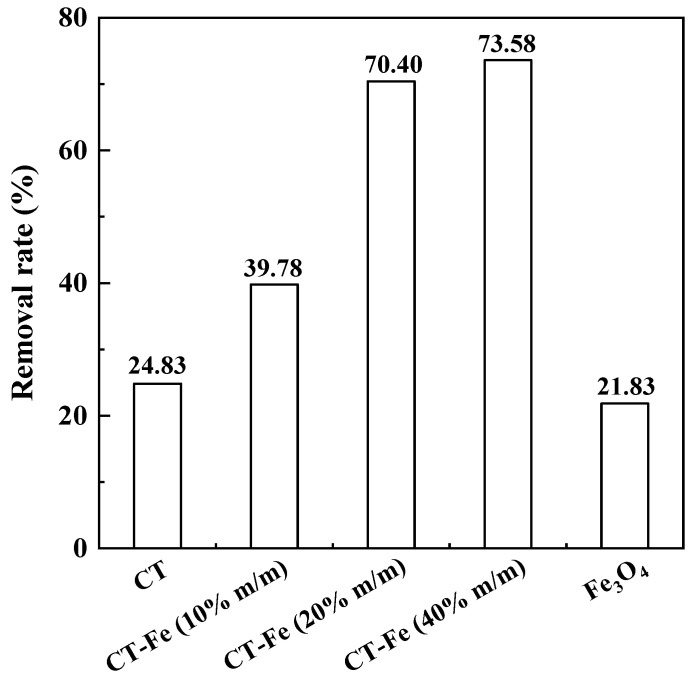
Effect of different ratios of Fe(II) on the removal rate of Cr(VI) by chitosan (CT) beads (CT-Fe 10% *w*/*w* for 0.35 g Fe(II); CT-Fe 20% *w*/*w* for 0.70 g Fe(II); CT-Fe 40% *w*/*w*) for 1.40 g Fe(II)). Reproduced with the permission from Ref. [14]. Copyright 2018 Elsevier.

Other factors, such as the contents of raw materials and preparation methods, could also influence Cr(VI) removal. Hosseinzadeh and Asl et al. prepared a novel poly(pyrrole)-based magnetic hydrogel nanocomposite using free-radical copolymerization of acrylic acid, pyrrole, and acrylamide monomers with the subsequent in situ synthesis of magnetic Fe_3_O_4_ nanoparticles [110]. The removal of Cr(VI) increased with the increase of the acrylic acid and acrylamide monomer concentrations due to the increased number of functional groups and the enhanced swelling capacity [110]. As the concentrations of acrylic acid and acrylamide monomers increased from 0.51 to 2.8 mol/L, the adsorption capacities of Cr(VI) increased from 43 to 122 and from 12 to 88 mg/g, respectively [110]. The presence of Fe_3_O_4_ nanoparticles, which increased the number of –OH groups and strengthened the overall porosity of the adsorbents, enhanced Cr(VI) removal [110]. Chitosan-stabilized nanostructured iron oxide (CT-Fe beads) was synthesized by the direct incorporation of FeCl_2_·4H_2_O into the chitosan gel. The magnetic iron oxide produced together with chitosan was identified as magnetite (Fe_3_O_4_). The removal rates of Cr(VI) by CT-Fe beads with different ratios of Fe(II) are shown in Figure 15. The removal rates by CT beads without Fe(II) and with 0.35, 0.7, and 1.4 g Fe(II) were 24.83%, 39.78%, 70.40%, and 73.58, respectively. The result was due to the metal active sites generated by the complexation of Fe with O and N atoms on the chitosan surface, which facilitated the reduction of Cr(VI). Because of the masking of active groups on the polymers, kaolin had shown adverse effects on Cr(VI) removal induced by polymeric adsorbents [127]. The preparation methods of the absorbents have shown a pronounced effect on Cr(VI) removal. For example, prolonging the polymerization time of PPy-coated sepiolite fiber could promote Cr(VI) removal due to a more complete reaction [26]. PPy synthesized through rapid mixing method generated both of bulk ppy and a homogenous ppy film, which were favorable for Cr(VI) removal, while the conventional chemical oxidation method only generated bulk ppy [28].

## 5. Regeneration of Absorbents

The reusability of the absorbents is an important factor that determines the application of plyometric adsorbents. The stronger the affinity of the adsorbent toward Cr(VI) is, the harder it is for of Cr(VI) to be desorbed. Various reagents, including H_2_O, 0.1 M thiourea, 0.1 M ethylenediaminetetraacetic acid (EDTA), 0.1 M HCl, and 0.1 M NaOH, were used to desorb Cr(VI) adsorbed on lignin/poly(vinyl alcohol) blend fibers [151]. The maximum desorption efficiency (~60%) was found in 1.0 M NaOH, while those of the others were lower than 20% [151]. Similar results were found for magnetic mesoporous carbon nanospheres, the Cr(VI) removal efficiencies decreased from 100% in the first cycle to 86.4 ± 7.1% in the second cycle and then to 69.7 ± 3.2% in the third cycle using 1.0 M NaOH as the regenerative reagent [154]. The application of HCl was also documented. The Cr(VI) removal efficiencies of PANI coated bacterial cellulose mat (PANi/BC mat) were ~100% in the first two cycles but 82% and 56% for the third and fourth cycles, respectively [56], in which 0.1 M HCl was used as the desorption agent [56]. The decline of Cr(VI) removal in the subsequent cycles could be explained by the partial damage of polymer structure by the strong acid/alkali treatment. Reagents with mild reactivities could be a more preferred choice. The adsorption capacity of hyper crosslinked mesoporous poly(ionic liquid)s (PVIm-6-SCD) toward Cr(VI) remained constant even after five-cycles of reuse [155]. During the process, Cr(VI) was desorbed using KBr via the ion-exchange mechanism [156]. Taken together, the development of mild reagents for desorbing Cr(VI) and the synthesis of polymers with strong chemical stabilities are necessary to promote the reuse of polymeric adsorbents.

## 6. Conclusions and Perspectives

Polymeric adsorbents, which are easy to assess functional groups, have shown robust performance in removing Cr(VI). Electron donating groups, amine/imine groups, carboxyl, hydroxyl, and phenol, facilitated the sorptive removal of Cr(VI) through reduction. Meanwhile, the presence of carboxyl and hydroxyl groups could enhance Cr(VI) removal through complexation or chelation mechanisms. Enrich in amino and imino groups, PPy- and PANI-based adsorbents are the most widely used polymeric adsorbents for Cr(VI) removal. However, their structural deterioration during the reduction process hindered their industrial application. Cellulose- and chitosan-based absorbents were environmentally friendly, while they were hard to be regenerated. Clay-based adsorbents were in low cost, while their adsorption capacities were need to be further improved. Nano-sized metals/metal oxides based polymeric adsorbents are in the emerging field with little available information on the Cr(VI) removal, thus more researched are needed. Carbon-based absorbents has shown robust performance on Cr(VI) removal, while they were non-selective and has relatively high price. Sorptive removal of Cr(VI) is generally endothermic, and the better performance of these polymeric adsorbents often occurred at higher temperature. The presence of anions and DOM could inhibited the Cr(VI) due to the competitive adsorption, while cations barely affected the process. Despite these progress, the industrial application of these novel materials are hindered by types of factors, such as the low efficiency, the high cost, the potential of second-pollution, and the complex of synthesis method. Following aspects are needed to bear in mind to synthesis more robust polymeric adsorbents and to promote their industrial application.

(1) Emphasize of detoxification

The toxicity of Cr(VI) is far greater than that of Cr(III) [157]. Since the adsorbed Cr(VI) ions could also have the potential to release from the adsorbents, reduction of Cr(VI) to Cr(III) is necessary. A sound strategy for Cr(VI) removal is reduction of Cr(VI) to Cr(III) and then removal of Cr(III) simultaneously to achieve the goal of detoxification and chromium removal. Oxygen-containing, nitrogen functional groups, and other electron-donating groups can act as the effective electron donors to reduce Cr(VI) [139,157]. Thus, the introduction of electron-donating groups to adsorbents could be effective for Cr(VI) detoxification.

(2) Highlights the removal of Cr(VI) at trace levels

Since Cr(VI) is harmful to human beings even in the extremely low concentration (i.e., 50 μg/L) [158], removal of Cr(VI) to satisfy this standard is necessary. The main advantage of adsorption method is capable of removing pollutants at relatively low levels when compared to that of chemical method. However, most of the researches were performed at relatively high levels of Cr(VI) (mg/L) (Table 1). It is well known that, sorptive removal of pollutants at relatively low levels is harder than that of at high levels due to weaker mass transfer. Despite the high removal rate and the maximum adsorption capacity of Cr(VI) being all high enough (Table 1), the residual Cr(VI) could also be higher than 50 μg/L. Thus, the polymeric material design should paid more emphasize on the removal efficiency of Cr(VI) at low concentrations to maximize the advantage of adsorption method.

(3) Development of absorbents for extensive reaction conditions

Works regarding Cr(VI) removal are mostly limited to batch experiments on a laboratory scale (Table 1). Meanwhile, the optimum pH values for most polymeric adsorbents are in the strong acidic conditions, which are hard for industrial application. Moreover, the treatment performances of these polymeric adsorbents were little investigated in real wastewater. Despite several factors are evaluated on Cr(VI) removal, they are far to reflect the realistic conditions of the wastewater. The fundamental consideration of material design is the practical application. Thus, the realistic scenarios of Cr(VI) removal should be more emphasized in the future work, and the materials design should to cope with the complexity of Cr(VI)-containing water matrix.

(4) Emphasizing of cost reduction

The regeneration and reusability of polymeric adsorbents are the two key factors determining the feasibility and sustainability of the adsorbents, which could not only save the treatment costs but also grantee the environmental friendly. Meanwhile, exploring of the natural polymers for Cr(VI) removal is also the cost-saving method. According to the previous report, the application of biomaterials in heavy metal removal as adsorbents declined by 20% of the investment cost, 36% of the operation cost and 28% of the entire treatment cost matched with several conventional technologies [100]. The application of natural raw materials such as clay or polysaccharides can minimize the cost of production of polymeric composites with a high contaminant removal capacity [91]. So the natural and low-cost raw materials for the production of the absorbents are recommended.

## Data Availability

Data sharing is not applicable.
